# 1-Benzoyl-3-chloro­azepan-2-one

**DOI:** 10.1107/S1600536809033510

**Published:** 2009-09-09

**Authors:** Hua-Quan Liu, Dong-Mei Fan, De-Cai Wang, Ping-Kai Ou-Yang

**Affiliations:** a State Key Laboratory of Materials-Oriented Chemical Engineering, College of Life Science and Pharmaceutical Engineering, Nanjing University of Technology, Xinmofan Road No. 5 Nanjing, Nanjing 210009, People’s Republic of China

## Abstract

In the crystal structure of the title compound, C_13_H_14_ClNO_2_, inter­molecular C—H⋯O inter­actions link the mol­ecules into a two-dimensional network.

## Related literature

For related structures, see: Tull *et al.* (1964 ▶); Largman *et al.* (1979 ▶). For ring-puckering parameters, see: Cremer & Pople (1975 ▶). For bond-length data, see: Allen *et al.* (1987 ▶).
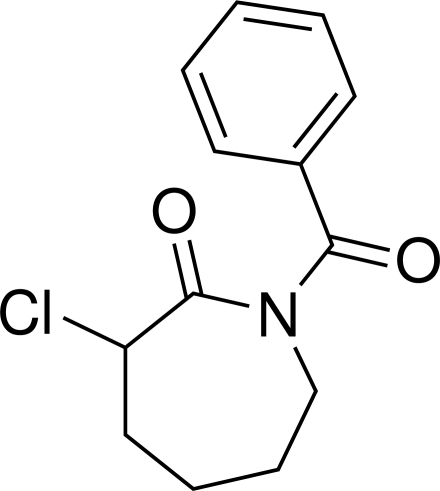


## Experimental

### 

#### Crystal data

C_13_H_14_ClNO_2_
*M* *_r_* = 251.70
Orthorhombic, 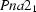

*a* = 19.564 (4) Å
*b* = 7.6500 (15) Å
*c* = 8.4050 (17) Å
*V* = 1257.9 (4) Å^3^
*Z* = 4
Mo *K*α radiation
μ = 0.29 mm^−1^
*T* = 294 K
0.30 × 0.20 × 0.10 mm

#### Data collection

Enraf–Nonius CAD-4 diffractometer
Absorption correction: ψ scan (North *et al.*, 1968 ▶) *T*_min_ = 0.917, *T*_max_ = 0.971
2413 measured reflections
1229 independent reflections
968 reflections with *I* > 2σ(*I*)
*R*_int_ = 0.027
3 standard reflections frequency: 120 min intensity decay: 1%

#### Refinement

*R*[*F* ^2^ > 2σ(*F* ^2^)] = 0.040
*wR*(*F* ^2^) = 0.109
*S* = 1.01
1229 reflections
155 parameters
1 restraint
H-atom parameters constrained
Δρ_max_ = 0.17 e Å^−3^
Δρ_min_ = −0.16 e Å^−3^
Absolute structure: Flack (1983 ▶), 1184 Friedel pairs
Flack parameter: 0.07 (12)

### 

Data collection: *CAD-4 Software* (Enraf–Nonius, 1989 ▶); cell refinement: *CAD-4 Software*; data reduction: *XCAD4* (Harms & Wocadlo, 1995 ▶); program(s) used to solve structure: *SHELXS97* (Sheldrick, 2008 ▶); program(s) used to refine structure: *SHELXL97* (Sheldrick, 2008 ▶); molecular graphics: *ORTEP-3 for Windows* (Farrugia, 1997 ▶) and *PLATON* (Spek, 2009 ▶); software used to prepare material for publication: *SHELXL97* and *PLATON*.

## Supplementary Material

Crystal structure: contains datablocks global, I. DOI: 10.1107/S1600536809033510/hk2754sup1.cif

Structure factors: contains datablocks I. DOI: 10.1107/S1600536809033510/hk2754Isup2.hkl

Additional supplementary materials:  crystallographic information; 3D view; checkCIF report

## Figures and Tables

**Table 1:** Hydrogen-bond geometry (Å, °)

*D*—H⋯*A*	*D*—H	H⋯*A*	*D*⋯*A*	*D*—H⋯*A*
C5—H5*A*⋯O1^i^	0.93	2.43	3.335 (6)	163
C12—H12*A*⋯O2^ii^	0.98	2.56	3.319 (5)	134

Symmetry codes: (i) 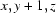; (ii) 
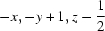
.

**Fig. 1.**
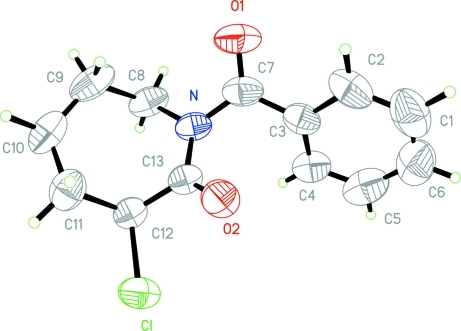
The molecular structure of the title molecule, with the atom-numbering scheme. Displacement ellipsoids are drawn at 30% probability level.

**Fig. 2.**
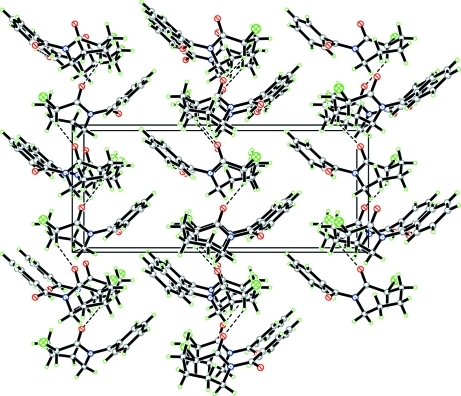
A partial packing diagram of the title compound. Hydrogen bonds are shown as dashed lines.

## supplementary crystallographic information

### Comment

*N*-substituted-3-chlorocaprolactams are used as medicines and as intermediate compounds for producing various organic chemicals. We report herein the crystal structure of the title compound.

In the molecule of the title compound, (Fig. 1), the bond lengths (Allen *et al.*,  1987) and angles are within normal ranges. Ring A (C1-C6) is, of course, planar, while the seven-membered ring B (N/C8-C13) is not planar, having total puckering amplitude, Q_T_, of 0.841 (2) Å (Cremer & Pople, 1975).

In the crystal structure, intermolecular C-H···O interactions (Table 1) link the molecules into a two dimensional network (Fig. 2), in which they may be efective in the stabilization of the structure.

### Experimental

The title compound was prepared according to a literature method (Tull *et al.*,  1964). The purity of the compound was checked by determining its melting point. It was characterized by recording its infrared and NMR spectra (Largman *et al.*,  1979). Crystals suitable for X-ray analysis were obtained from slow evaporation of an ethanol solution.

### Refinement

H atoms were positioned geometrically with C-H = 0.93, 0.98 and 0.97 Å for aromatic, methine and methylene H atoms, respectively, and constrained to ride on their parent atoms, with U_iso_(H) = 1.2U_eq_(C).

### Crystal data

**Table:**

C_13_H_14_ClNO_2_	*F*(000) = 528
*M**_r_* = 251.70	*D*_x_ = 1.329 Mg m^−^^3^
Orthorhombic, *P**n**a*2_1_	Mo *K*α radiation, λ = 0.71073 Å
Hall symbol: P 2c -2n	Cell parameters from 25 reflections
*a* = 19.564 (4) Å	θ = 10–13°
*b* = 7.6500 (15) Å	µ = 0.29 mm^−^^1^
*c* = 8.4050 (17) Å	*T* = 294 K
*V* = 1257.9 (4) Å^3^	Block, colorless
*Z* = 4	0.30 × 0.20 × 0.10 mm

### Data collection

**Table:**

Enraf–Nonius CAD-4 diffractometer	968 reflections with *I* > 2σ(*I*)
Radiation source: fine-focus sealed tube	*R*_int_ = 0.027
graphite	θ_max_ = 25.3°, θ_min_ = 2.1°
ω/2θ scans	*h* = −23→23
Absorption correction: ψ scan (North *et al.*, 1968)	*k* = 0→9
*T*_min_ = 0.917, *T*_max_ = 0.971	*l* = 0→10
2413 measured reflections	3 standard reflections every 120 min
1229 independent reflections	intensity decay: 1%

### Refinement

**Table:**

Refinement on *F*^2^	Hydrogen site location: inferred from neighbouring sites
Least-squares matrix: full	H-atom parameters constrained
*R*[*F*^2^ > 2σ(*F*^2^)] = 0.040	*w* = 1/[σ^2^(*F*_o_^2^) + (0.068*P*)^2^] where *P* = (*F*_o_^2^ + 2*F*_c_^2^)/3
*wR*(*F*^2^) = 0.109	(Δ/σ)_max_ < 0.001
*S* = 1.01	Δρ_max_ = 0.17 e Å^−^^3^
1229 reflections	Δρ_min_ = −0.16 e Å^−^^3^
155 parameters	Extinction correction: *SHELXL97* (Sheldrick, 2008), Fc^*^=kFc[1+0.001xFc^2^λ^3^/sin(2θ)]^-1/4^
1 restraint	Extinction coefficient: 0.020 (3)
Primary atom site location: structure-invariant direct methods	Absolute structure: Flack (1983), 1184 Friedel pairs
Secondary atom site location: difference Fourier map	Flack parameter: 0.07 (12)

### Special details

**Table:**

Geometry. All e.s.d.'s (except the e.s.d. in the dihedral angle between two l.s. planes) are estimated using the full covariance matrix. The cell e.s.d.'s are taken into account individually in the estimation of e.s.d.'s in distances, angles and torsion angles; correlations between e.s.d.'s in cell parameters are only used when they are defined by crystal symmetry. An approximate (isotropic) treatment of cell e.s.d.'s is used for estimating e.s.d.'s involving l.s. planes.
Refinement. Refinement of *F*^2^ against ALL reflections. The weighted *R*-factor *wR* and goodness of fit *S* are based on *F*^2^, conventional *R*-factors *R* are based on *F*, with *F* set to zero for negative *F*^2^. The threshold expression of *F*^2^ > σ(*F*^2^) is used only for calculating *R*-factors(gt) *etc*. and is not relevant to the choice of reflections for refinement. *R*-factors based on *F*^2^ are statistically about twice as large as those based on *F*, and *R*- factors based on ALL data will be even larger.

### Fractional atomic coordinates and isotropic or equivalent isotropic displacement parameters (Å^2^)

**Table:**

	*x*	*y*	*z*	*U*_iso_*/*U*_eq_	
Cl	0.11953 (5)	0.57305 (14)	0.24751 (14)	0.0715 (4)	
O1	−0.1309 (2)	0.1107 (4)	0.1382 (6)	0.1086 (15)	
O2	−0.00941 (14)	0.4326 (3)	0.3449 (4)	0.0675 (9)	
N	−0.03396 (16)	0.2690 (4)	0.1259 (4)	0.0536 (8)	
C1	−0.2327 (2)	0.5048 (9)	0.3917 (8)	0.0978 (18)	
H1A	−0.2664	0.4839	0.4674	0.117*	
C2	−0.1934 (2)	0.3696 (7)	0.3412 (7)	0.0795 (14)	
H2A	−0.2007	0.2575	0.3804	0.095*	
C3	−0.14219 (19)	0.4004 (5)	0.2300 (6)	0.0595 (10)	
C4	−0.1317 (2)	0.5660 (5)	0.1702 (6)	0.0682 (12)	
H4A	−0.0972	0.5876	0.0968	0.082*	
C5	−0.1740 (3)	0.6992 (7)	0.2223 (7)	0.0935 (17)	
H5A	−0.1688	0.8113	0.1811	0.112*	
C6	−0.2244 (3)	0.6671 (9)	0.3359 (9)	0.0995 (19)	
H6A	−0.2520	0.7577	0.3725	0.119*	
C7	−0.1033 (2)	0.2489 (5)	0.1659 (5)	0.0664 (11)	
C8	−0.0071 (2)	0.1496 (5)	0.0028 (5)	0.0667 (12)	
H8A	0.0183	0.2172	−0.0749	0.080*	
H8B	−0.0452	0.0950	−0.0518	0.080*	
C9	0.0390 (3)	0.0080 (5)	0.0694 (6)	0.0801 (14)	
H9A	0.0200	−0.0327	0.1693	0.096*	
H9B	0.0392	−0.0901	−0.0038	0.096*	
C10	0.1113 (3)	0.0645 (6)	0.0972 (7)	0.0812 (15)	
H10A	0.1315	0.0921	−0.0052	0.097*	
H10B	0.1362	−0.0344	0.1402	0.097*	
C11	0.1229 (2)	0.2193 (5)	0.2068 (5)	0.0695 (12)	
H11A	0.1712	0.2481	0.2068	0.083*	
H11B	0.1105	0.1859	0.3143	0.083*	
C12	0.08196 (19)	0.3832 (4)	0.1600 (4)	0.0524 (9)	
H12A	0.0820	0.3955	0.0439	0.063*	
C13	0.00874 (18)	0.3694 (4)	0.2188 (4)	0.0493 (9)	

### Atomic displacement parameters (Å^2^)

**Table:**

	*U*^11^	*U*^22^	*U*^33^	*U*^12^	*U*^13^	*U*^23^
Cl	0.0725 (6)	0.0733 (7)	0.0686 (7)	−0.0204 (5)	0.0016 (6)	−0.0118 (7)
O1	0.103 (3)	0.0654 (18)	0.158 (5)	−0.0333 (17)	−0.006 (3)	−0.010 (3)
O2	0.0634 (16)	0.086 (2)	0.0532 (18)	−0.0064 (14)	0.0031 (14)	−0.0231 (17)
N	0.0711 (19)	0.0486 (16)	0.0411 (16)	−0.0102 (15)	−0.0084 (15)	−0.0033 (15)
C1	0.057 (3)	0.139 (5)	0.098 (4)	−0.001 (3)	−0.003 (3)	−0.006 (4)
C2	0.059 (2)	0.096 (3)	0.083 (3)	−0.016 (2)	−0.009 (3)	0.022 (3)
C3	0.0542 (19)	0.068 (2)	0.056 (2)	−0.0110 (18)	−0.015 (2)	0.007 (2)
C4	0.083 (3)	0.060 (2)	0.061 (3)	−0.007 (2)	−0.014 (2)	0.008 (2)
C5	0.120 (4)	0.068 (3)	0.093 (4)	0.007 (3)	−0.040 (4)	0.004 (3)
C6	0.066 (3)	0.116 (5)	0.117 (5)	0.027 (3)	−0.029 (4)	−0.027 (4)
C7	0.078 (3)	0.057 (2)	0.064 (3)	−0.016 (2)	−0.020 (2)	0.006 (2)
C8	0.104 (3)	0.055 (2)	0.041 (2)	−0.008 (2)	−0.009 (2)	−0.011 (2)
C9	0.141 (4)	0.047 (2)	0.052 (3)	0.003 (3)	0.000 (3)	−0.006 (2)
C10	0.115 (4)	0.056 (3)	0.073 (3)	0.022 (2)	0.009 (3)	0.007 (3)
C11	0.083 (3)	0.066 (2)	0.059 (3)	0.014 (2)	−0.006 (2)	0.005 (2)
C12	0.065 (2)	0.052 (2)	0.0396 (19)	−0.0011 (17)	0.0015 (19)	−0.0028 (17)
C13	0.062 (2)	0.0466 (18)	0.039 (2)	−0.0036 (16)	−0.0013 (17)	−0.0010 (17)

### Geometric parameters (Å, °)

**Table:**

Cl—C12	1.786 (4)	C5—H5A	0.9300
O1—C7	1.210 (5)	C6—H6A	0.9300
O2—C13	1.218 (5)	C8—C9	1.517 (6)
N—C7	1.406 (5)	C8—H8A	0.9700
N—C8	1.477 (5)	C8—H8B	0.9700
N—C13	1.377 (5)	C9—C10	1.497 (7)
C1—C6	1.338 (8)	C9—H9A	0.9700
C1—C2	1.357 (7)	C9—H9B	0.9700
C1—H1A	0.9300	C10—C11	1.518 (7)
C2—C3	1.390 (6)	C10—H10A	0.9700
C2—H2A	0.9300	C10—H10B	0.9700
C3—C4	1.378 (5)	C11—C12	1.539 (5)
C3—C7	1.487 (6)	C11—H11A	0.9700
C4—C5	1.384 (7)	C11—H11B	0.9700
C4—H4A	0.9300	C12—C13	1.519 (5)
C5—C6	1.394 (8)	C12—H12A	0.9800
			
C7—N—C8	116.3 (3)	H8A—C8—H8B	107.7
C13—N—C7	120.7 (3)	C10—C9—C8	114.4 (4)
C13—N—C8	121.7 (3)	C10—C9—H9A	108.7
C6—C1—C2	121.9 (6)	C8—C9—H9A	108.7
C6—C1—H1A	119.1	C10—C9—H9B	108.7
C2—C1—H1A	119.1	C8—C9—H9B	108.7
C1—C2—C3	119.4 (5)	H9A—C9—H9B	107.6
C1—C2—H2A	120.3	C9—C10—C11	117.5 (4)
C3—C2—H2A	120.3	C9—C10—H10A	107.9
C4—C3—C2	120.5 (4)	C11—C10—H10A	107.9
C4—C3—C7	120.5 (4)	C9—C10—H10B	107.9
C2—C3—C7	118.7 (4)	C11—C10—H10B	107.9
C3—C4—C5	118.2 (5)	H10A—C10—H10B	107.2
C3—C4—H4A	120.9	C10—C11—C12	113.8 (4)
C5—C4—H4A	120.9	C10—C11—H11A	108.8
C4—C5—C6	120.6 (5)	C12—C11—H11A	108.8
C4—C5—H5A	119.7	C10—C11—H11B	108.8
C6—C5—H5A	119.7	C12—C11—H11B	108.8
C1—C6—C5	119.3 (5)	H11A—C11—H11B	107.7
C1—C6—H6A	120.3	C13—C12—C11	110.6 (3)
C5—C6—H6A	120.3	C13—C12—Cl	108.1 (2)
O1—C7—N	118.7 (4)	C11—C12—Cl	110.0 (3)
O1—C7—C3	121.5 (4)	C13—C12—H12A	109.4
N—C7—C3	119.7 (3)	C11—C12—H12A	109.4
N—C8—C9	113.3 (3)	Cl—C12—H12A	109.4
N—C8—H8A	108.9	O2—C13—N	122.5 (4)
C9—C8—H8A	108.9	O2—C13—C12	122.0 (3)
N—C8—H8B	108.9	N—C13—C12	115.2 (3)
C9—C8—H8B	108.9		
			
C6—C1—C2—C3	1.1 (8)	C13—N—C8—C9	60.9 (5)
C1—C2—C3—C4	−0.7 (7)	C7—N—C8—C9	−106.4 (4)
C1—C2—C3—C7	−175.1 (5)	N—C8—C9—C10	−82.1 (5)
C2—C3—C4—C5	−0.8 (7)	C8—C9—C10—C11	57.4 (6)
C7—C3—C4—C5	173.5 (4)	C9—C10—C11—C12	−53.5 (6)
C3—C4—C5—C6	2.0 (7)	C10—C11—C12—C13	80.6 (5)
C2—C1—C6—C5	0.1 (9)	C10—C11—C12—Cl	−160.0 (3)
C4—C5—C6—C1	−1.6 (8)	C7—N—C13—O2	6.3 (5)
C13—N—C7—O1	−145.6 (5)	C8—N—C13—O2	−160.5 (3)
C8—N—C7—O1	21.9 (6)	C7—N—C13—C12	−178.7 (3)
C13—N—C7—C3	38.8 (5)	C8—N—C13—C12	14.5 (5)
C8—N—C7—C3	−153.7 (4)	C11—C12—C13—O2	94.8 (4)
C4—C3—C7—O1	−136.6 (5)	Cl—C12—C13—O2	−25.7 (4)
C2—C3—C7—O1	37.8 (7)	C11—C12—C13—N	−80.3 (4)
C4—C3—C7—N	38.9 (6)	Cl—C12—C13—N	159.2 (3)
C2—C3—C7—N	−146.7 (4)		

### Hydrogen-bond geometry (Å, °)

**Table:**

*D*—H···*A*	*D*—H	H···*A*	*D*···*A*	*D*—H···*A*
C5—H5A···O1^i^	0.93	2.43	3.335 (6)	163
C12—H12A···O2^ii^	0.98	2.56	3.319 (5)	134

Symmetry codes: (i) *x*, *y*+1, *z*; (ii) −*x*, −*y*+1, *z*−1/2.
